# A systematic review of HIV screening programs conducted in pediatric emergency departments in the United States

**DOI:** 10.1186/s12873-022-00633-5

**Published:** 2022-05-06

**Authors:** Lynn Bi, Rachel E. Solnick, Roland C. Merchant

**Affiliations:** 1 Department of Emergency Medicine, Brigham and Women’s Hospital, Harvard Medical School , Boston, MA USA; 2 Department of Emergency Medicine, Icahn School of Medicine at Mount Sinai, 1 Gustave L. Levy Place, New York, NY 10029 USA

**Keywords:** HIV testing, Pediatric emergency medicine, Systematic review, United States, Diagnostic screening programs, Adolescent, HIV

## Abstract

**Background:**

We conducted a systematic review of studies published in peer-reviewed journals on HIV screening programs conducted in pediatric emergency departments (PEDs) in the United States (US) with the objective of describing the methods, testing yields and challenges in these programs.

**Methods:**

We searched for full-text, English-language, original research articles focused on the conduct, development, initiation or implementation of any HIV screening program in a US PED through eight online databases (Pubmed (MEDLINE), Scopus, Embase, Cochrane, Web of Science, CINAHL, PsycInfo and Google Scholar) from their inception through July 2020. We also searched for articles on the websites of thirteen emergency medicine journals, 24 pediatric and adolescent health journals, and ten HIV research journals, and using the references of articles found through these searches. Data on HIV testing program components and yield of testing was extracted by one investigator independently and verified by a second investigator. Each program was summarized and critiqued.

**Results:**

Of the eight articles that met inclusion criteria, most involved descriptions of their HIV testing program, except for one that was focused on quality improvement of their program. Five described an opt-in and three an opt-out approach to HIV screening. Programs differed greatly by type of HIV test utilized and who initiated or performed testing. There were large variations in the percentage of patients offered (4.0% to 96.7%) and accepting (42.7% to 86.7%) HIV testing, and HIV seropositivity in the studies ranged from 0 to 0.6%. Five of the eight studies reported an HIV seropositivity greater than 0.1%, above Centers for Disease Control and Prevention recommended threshold for testing in a healthcare setting.

**Conclusions:**

The studies illustrate opportunities to further optimize the integration of HIV screening programs within US PEDs and reduce barriers to testing, improve efficiency of testing results and increase effectiveness of programs to identify cases. Future research should focus on advancing the methodology of screening programs beyond feasibility studies as well as conducting investigations on their implementation and longer-term sustainability.

**Supplementary Information:**

The online version contains supplementary material available at 10.1186/s12873-022-00633-5.

## Background

Adolescents 13–19 years-old and young adults 20–24 years-old accounted for 0.5% and 2.6%, respectively, of the estimated 1.1 million people living with the human immunodeficiency virus (HIV) in the United States (US) in 2018 [1]. However, approximately 21% of new US HIV diagnoses in 2018 occurred among 13 to 24 year-olds, and 20 to 24 year-olds had with the second highest rate of new diagnoses among all age groups [1]. Furthermore as of 2018, almost 45% of 13–24-year-old HIV-infected adolescents and young adults were unaware of their HIV status, which was the highest rate of undiagnosed HIV infections among all age groups [2]. Among US high school students surveyed in 2017, only 9.3% had ever been tested for HIV (not including blood donations), and notably only 13.2% of those who reported having sex with the opposite sex and 20.2% with the same sex had ever been tested [3]. These statistics underscore the need for HIV testing among adolescents as well as young adults so that they can be linked to care if HIV infected and receive preventive services if at continued or future risk of acquiring HIV.

To increase the number of adolescents aware of their HIV infection, the American Academy of Pediatrics (AAP) recommends that: (1) adolescents should be tested for at least once by 16 to 18 years of age in health care settings where the prevalence of HIV in the adolescent patient population is more than 0.1%: (2) adolescents living in lower HIV prevalence settings who are having sex or at risk for HIV because of drug use should be tested; (3) higher risk adolescents should be tested annually for HIV; (4) those who are being tested for sexually transmitted infections (STIs) should be tested for HIV during the same visit; and (5) emergency departments (EDs) and urgent care areas in higher prevalence areas should implement routine HIV testing. The AAP recommendations echo the 2006 US Centers for Disease Control and Prevention (CDC) recommendations for expanding HIV screening in all healthcare settings, including EDs, for patients 13 to 64 years-old [4]. The United States Preventive Services Task Force (USPSTF) recommended in 2019 that clinicians conduct HIV screening regardless of risk at least once for patients 15 to 65 years-old, and screening based on risk for adolescents < 15 years-old [5].

Despite the aforementioned recommendations addressing HIV testing for adolescents in US EDs, HIV screening and diagnostic testing are performed infrequently in this setting. Of the over 10 million visits by 13–19-years-olds to US EDs from 2009–2017, HIV testing was conducted in only 0.53% of visits [6]. Research indicates practice and knowledge barriers to HIV testing in US pediatric EDs (PEDs). Of pediatric emergency medicine (PEM) groups from seven metropolitan areas in the US surveyed in 2012, 51% reported having an HIV testing guideline in their ED [7]. In an unrelated 2012 survey of attending PEM physicians, only 28% correctly identified the age group recommended by CDC for HIV screening [8]. The consequences of lack of pediatric ED HIV testing were illustrated in an evaluation of young adults diagnosed with HIV through a screening program at the Grady Hospital adult ED [9]. Of the 193 young adults, 38 had 109 PED visits in the ten years prior to their HIV diagnosis, reflecting missed opportunities for testing and prevention of future infections. These accumulated findings demonstrate that there are opportunities to improve and expand pediatric ED HIV testing.

To better understand the current status of research on HIV screening in US PEDs, we conducted a systematic review of existing studies on HIV screening initiatives in US PEDs. Our objective was to review the types of programs conducted to date, their approaches, research methodologies and yields of testing. From this review, we aimed to provide a perspective on what research might assist in guiding expansion of HIV screening in US PEDs.

## Methods

The Preferred Reporting Items for Systematic Reviews and Meta-Analyses (PRISMA) guidelines were followed for this systematic review [10].

### Information sources and search strategy

We initially performed a search for research articles using the online databases PubMed (MEDLINE), Scopus, Embase, Cochrane, Web of Science, CINAHL, PsycInfo and Google Scholar from their inception through July 2020. The search was repeated prior to acceptance of the article in January 2022. Search terms centered around all applicable controlled vocabularies and key terms related to “pediatric emergency department” and “HIV screening.” We subsequently conducted manual searches of the websites of thirteen emergency medicine journals, 24 pediatric and adolescent health journals, and ten HIV research journals for additional relevant articles (Supplement). We reviewed past issues of each journal through a search on their respective websites. These journals were selected by the authors from the listings for these types of journals on PubMed (MEDLINE). Hand searches through references of articles found through these searches also were performed.

### Inclusion/exclusion criteria

Articles included in this systematic review were full-text, English-language, original research articles focused on the conduct, development, initiation or implementation of any HIV screening program in a US PED. Studies were excluded if the HIV screening program was based in an adult or combined pediatric and adult ED, adolescent or HIV clinic, or any other primary care settings. Abstracts, case studies, editorials, opinion pieces, commentaries or review articles were not included. Peer-reviewed brief reports and research letters were included. No time restrictions were applied.

### Data collection and analysis

Citations from the initial database and manual searches were collected. Duplicate citations were removed, and the titles and abstracts of each source were screened based on the inclusion and exclusion criteria. Full-text articles were then examined for their eligibility by the two reviewers, who reached a consensus regarding the final set of articles included for data abstraction and review. With these articles, the reviewers created a form based on the work by Lyons, et al. [11] to guide extraction of key information on the setting and components of each HIV testing program (e.g., personnel involved, testing methodologies, consent obtained), program inclusion/exclusion criteria and the yield of the program (e.g., the number of program eligible patients; patients who were offered HIV testing and/or counselling, accepted testing, were tested, tested positive for HIV, and linked to care was extracted). A meta-analysis was not possible due to the heterogeneity in the methodology of the studies and their outcomes; therefore, a qualitative analysis was performed.

## Results

### Overview

**Fig.1:**
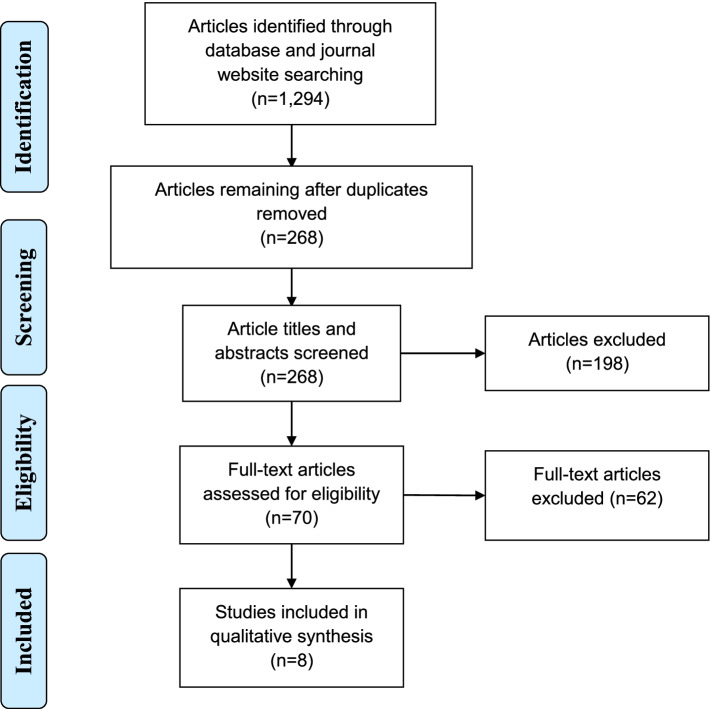
Preferred Reporting Items for Systematic Reviews and Meta-Analyses (PRISMA) diagram of article searches through inclusion

Eight studies were included in this systematic review based on study criteria for adolescent and young adult HIV screening programs implemented in US pediatric ED settings. (Figure 1) The ages of the patients included differed among the eight programs described, but all ranged between 13 and 24 years-old. (Table 1) Of the eight programs, five articles described an opt-in and three an opt-out approach to HIV screening, although details were not clearly described for all programs. Studies varied widely on the percentage of patients offered (4.0% to 96.7%) and accepting (42.7% to 86.7%) HIV testing. HIV seropositivity in the studies ranged from 0 to 0.6%. Five of the eight studies reported an HIV seropositivity greater than 0.1%, above the CDC recommended threshold for testing in a healthcare setting.

**Table 1:** Pediatric emergency department HIV screening studies data summary

Setting			HIV Consent				Testing assay		HIV Testing Program		
**Year**	**Geographic location**	**ED volume (Visits/year)**	**Consent**	**Default assumption of patient willingness**	**Patient indication of willingness**	**Parental involvement**	**Assay**	**Additional test (Confirmatory test)**	**Hours of coverage**	**Program Staffing**	**Program details**
April 1995—March 1997	Milwaukee, WI	75,000	Voluntary	Opt-in	Written Consent	No parental consent > 13 years	Phlebotomized samples for ELISA	Western blot		ED nurse	Program improvement initiative; follow-up pre- vs. post-program intervention evaluated
November 2003—May 2004	Boston, MA	25,000	Voluntary	Opt-in	Written Consent > 18 years	Assent & parent/ guardian permission for 15–17 years	OraSure (Orasure Technologies, Inc.) oral fluid sampling; Testing performed by state laboratory after the patient visit	Western blot	Mondays-Fridays, noon-10 pm	One research assistant	
September 2003—August 2006	Philadelphia, PA	70,000	Voluntary	Opt-in	Verbal consent for sexual health counseling; written consent for HIV testing	Not required per state law	OraSure (Orasure Technologies, Inc.) oral fluid sampling; Testing performed by an offsite laboratory		32 h/week	Health Educator	Program primarily provided sexual health counseling (30 min); testing offered after counseling. Patients presenting with possible sexually transmitted diseases and those referred by ED providers received priority for counseling
March 2008—October 2008	Memphis, TN	90,000	Implied	Opt-out	General consent & information sheet on HIV		Oraquick Advance Rapid HIV Antibody 1/2 test (OraSure Technologies, Inc) using oral fluid sampling	Western blot		Nurses	
October 2009—December 2009	Newark, NJ	35,000	Voluntary	Opt-in	Written Consent		Clearview HIV 1/2 STAT-PAK (Chembio Diagnostics Systems, Inc.) rapid HIV fingerstick testing	Western blot	Every day, 24 h / day	Offer testing: PED clinical providers and department of health HIV counselors; Perform testing: HIV counselors	
March 2009-February 2011	Washington, DC	126,000 combined (2 PEDs)	Voluntary	Opt-out	Verbal consent		Oraquick Advance Rapid HIV 1/2 Antibody Test (OraSure Technologies, Inc.) using oral fluid sampling	Western blot		Grant-funded personnel at ED1 and ED personnel at ED2 performed testing	
January 2012-December 2016	Cincinnati, OH	89,000	Voluntary	Opt-out	Verbal consent		Oraquick Advance Rapid HIV 1/2 Antibody Test (OraSure Technologies, Inc) using oral fluid sampling	4^th^ generation antigen/antibody testing	Every day, 24 h / day	PED clinical providers	
June 2019-October 2019	Atlanta, GA	60,000	Voluntary	Opt-out	Verbal consent		Architect HIV Ag/Ab Combo Test (Abbott Laboratories, Inc.); Fingerstick or phlebotomy sampling		40–60 h per week	Study investigators	

Setting			Postresults Communication and Methods	Patient Selection Strategies and Criteria			HIV Testing and Yield							Result Notification/ Linkage	
**Year**	**Geographic location**	**ED volume (Visits/year)**	**Post results notification and referral**	**Age**	**Screening**	**Exclusion criteria**	**Population count (Eligible by age)**	**Eligible (by inclusion/exclusion criteria)**	**Offered**	**Accepting HIV testing**	**Tested (if different number than accepted)**	**Reactive**	**Confirmed positive (n/tested %)**	**Result notification**	**Linkage to HIV care**
April 1995—March 1997	Milwaukee, WI	75,000	ED nurse was responsible for arranging follow-up of HIV test results	12–18 years old	Targeted: presenting for possible STD		Pre-program: 490 STD visits; Post-program: 372 STD visits				Pre-program: 18% HIV tested Post-program: 27% HIV testing		4 ELISA positive HIV tests; 3 indeterminate and 1 negative Western blot	Pre-program:8.1% notified of HIV test results Post-program: 57% notified of test results	2
November 2003—May 2004	Boston, MA	25,000	Follow-up of testing results was scheduled two weeks post-testing at an urgent care facility. The appointments of those who did not follow-up were not rescheduled	15–21 years old	Nontargeted	Tested for sexually transmitted diseases/HIV in past month; known HIV-positive status; non English- or Spanish-speaking; critically ill; intoxicated or presented with a psychiatric illness; university students or hospital employees; receiving care in common (open, less private) treatment areas of the ED; report being not “sexually experienced” if 15–17 years-old; presented to ED solely for HIV testing	1,749 Age-eligible; 1,202 Approached	791	765 Offered (96.7%)	464 Accepted (60.7%)	459		1 (0.2%)		1
September 2003—August 2006	Philadelphia, PA	70,000	Test results given within two weeks at a follow-up appointment	14–24 years old	Nontargeted	Victims of child abuse; “significantly developmentally delayed”; medically “unstable” per ED treatment team	32,121		1,287 Offered counseling (4%)	318 Accepted HIV testing and Tested (49.4%) (643 Accepted counseling (50.0%))			2 (0.6%)		2
March 2008—October 2008	Memphis, TN	90,000	Follow-up for testing appointment given 7–10 days post-testing at an adolescent HIV clinic	13–18 years old	Nontargeted	Critically-ill or injured		5,399	2,002 Approached (37.1%)	1,735 Accepted and Tested (86.7%)			1 (0.06%)		1
October 2009—December 2009	Newark, NJ	35,000		13–20 years old	Nontargeted	Critically ill; altered mental status; tested for HIV within past 3 months	2,645		300 Offered (11.3%)	224 Accepted (74.7%)	213		0 (0.0%)		NA
March 2009-February 2011	Washington, DC	126,000 combined (2 PEDs)	Patients whose rapid HIV test was positive received individual counseling with the case manager in the ED and a follow-up appointment 48–72 h after their PED visit at the specialized adolescent HIV services in the same medical center	13–24 years old	Nontargeted	Documented HIV test in the ED within the past six months (unless identified as high-risk)		8,528	8,519 (visits) Offered (77.5%)	6,184 Accepted (72.6%)	5,764	12	8 (.14%)		8
January 2012-December 2016	Cincinnati, OH	89,000	Patients ≤ 18 years old were scheduled to follow up in 7–10 days at the HIV clinic or primary care, and patients older than 18 years old were scheduled for a follow-up at the adult HIV clinic		Targeted: presenting for possible STD	Denied ever being sexually active or were being evaluated for concerns of sexual assault/abuse			4,378 Offered			14	11 (0.25%) 8 newly positive; 3 previously positive		11
June 2019-October 2019	Atlanta, GA	60,000		13–18 years old		Critically ill; unable to consent due to developmental delays or impaired mental status; deemed ineligible by their attending provider; previously diagnosed with HIV	1,307	1,061	806 Offered (76%)	344 Accepted (42.7%)			1 (0.3%)		1

### Summaries and critiques of the eight pediatric ED HIV testing studies

#### Beckman, et al. Wisconsin Medical Journal, 2002 [12]

##### Program description

This study involved the institution of a quality improvement initiative for HIV testing in a PED. The initiative was designed to increase patient receipt of their HIV test results and to overcome clinician reluctance to perform HIV testing due to concerns about responsibility for follow-up arrangements [12]. The target population for HIV testing in this PED was adolescents undergoing an STI evaluation. For this initiative, a PED nurse was tasked to contact all patients who were tested for HIV and notify them of their results. The initiative resulted in an increase in HIV testing (27% vs. 18%) and receipt of results (57% vs. 8.1%).

##### Critique

This program demonstrated how simple initiatives designed to overcome barriers can improve HIV testing efforts in a PED. Limitations of the program include its narrow scope (only patients with suspected STIs), few numbers of patients tested for HIV as a comparison of the PED’s patient volume, low rates of testing and follow-up. The analysis was limited due to the common limitations of retrospective medical record reviews, including the completeness, quality and need for interpretation of the documentation available to the study.

#### Mehta, et al. Academic Emergency Medicine, 2007 [13]

##### Program description

A research assistant identified and approached age-eligible patients after reviewing the daily ED log and triage notes, confirmed their study eligibility, and obtained consent [13]. Patients underwent oral fluid sampling with testing performed at a state laboratory separate from the PED. The most common reasons reported by patients for refusing testing were having recently tested for HIV in the previous one to six months and not perceiving a need for testing.

##### Critique

The study demonstrated feasibility of implementing this type of program in an urban PED and successfully identified and linked to care one HIV-infected patient who had not been HIV tested during the prior three years. Limitations of this program include its short duration (seven months), performance at a single site, restrictions of when testing was available, losses of testing opportunities due to study eligibility requirements, patients missed who were eligible during testing periods, and inability to perform HIV testing, provide results, and link HIV-infected patients to care during the ED visit. The consent process, particularly the need for parent or guardian permission for minor patients, posed significant barriers for HIV testing and study enrollment, and accounted for more than one-third of all ineligible cases. Approximately 9% of patients approached did not have a parent/guardian present with them; eleven did not want to ask their parents or guardian for consent; and one patient’s parent or guardian refused to give permission. The authors believed that the additional requirements involved in the context of conducting a research study and privacy concerns (treatment in common/open areas) also were barriers, although these aspects were not measured.

#### Mollen, et al. AIDS Patient Care and STDs, 2008 [14]

##### Program description

This sexual health counseling program sponsored by a grant from a local health department offered HIV testing following a 30-min counseling session with a health educator [14]. The health educator identified eligible patients through the ED log or by referral by the patient’s PED health care providers, preferentially approaching those presenting with a possible STI. A private room in the hospital was available for counseling and testing for patients awaiting discharge. The most commonly reported reasons for refusing counseling were being in too much pain or a lack of interest in the program.

##### Critique

The HIV screening program successfully identified and linked to care two HIV-infected patients and demonstrated success in integrating a sexual health counseling program with HIV testing using a dedicated health educator in a PED. The ability of patients to participate in the program without parental/guardian approval was a strength of the program. Limitations included that the program: relied on the availability and external funding of a health educator, made testing available only for the limited period of time when the health educator was present, involved a lengthy time required for counseling and a single test provider which reduced the number of patients who could be tested, targeted higher risk patients (predominately STI visits), preferentially involved only females (75.1% of program recipients), and did not make testing results during the ED visit (42% did not receive their HIV test result).

#### Minnear, et al. Pediatrics, 2009 [15]

##### Program description

In this program, eligible patients were provided with a 1-page HIV screening informational handout after registration and prior to being triaged [15]. ED nurses informed these patients that HIV screening was performed routinely for all adolescents in the ED, and asked them if they would rather not be tested. Oral fluid, rapid HIV testing was performed, although the article does not specify by whom and where the test was conducted. At five months after the testing program was initiated, efforts to increase the proportion of patients approached for HIV testing were implemented, including displaying wall posters, mounting computer monitor stickers, and instituting a visual computerized prompt was added to the electronic chart to remind staff members to offer screening. These efforts increased the proportions of patients approached from 28.9% to 54.3%. Testing acceptance was higher for adolescents 15 years-old and older than younger adolescents, but did not differ by race or gender. Most common reasons for declining testing were having been tested previously and perceiving themselves as not at risk.

##### Critique

Uptake of HIV testing was high for this opt-out screening program, and rapid HIV testing allowed for receipt of testing before discharge. However, the program occurred over a short time period (approximately 7.5 months), and test kits were provided from the state department of health, which likely limits sustainability. Furthermore, although testing was intended to be non-targeted, the study authors discovered that prior to the implementation of computerized testing prompts, older, nonwhite, female patients were more likely to be approached for testing, and after prompt initiation, older adolescents still were approached more often. In addition, although the interventions to increase those approached was successful, 62.9% of those eligible for testing were not approached. As an explanation for the low approach proportion, the study authors noted that lack of attendance at training sessions was high among nursing staff.

#### Hack, et al. Pediatric Emergency Care, 2013 [16]

##### Program description

Eligible patients were approached for HIV screening in triage or in the PED [16]. A paper form documenting the patients’ age, sex, race and testing acceptance or refusal was included in each patient’s chart, and was collected by PED nurses and physicians. After signed consent forms were obtained, pretest counseling, rapid HIV fingerstick testing and posttest counseling was performed by HIV counselors supplied by the state’s department of health. Acceptance of testing did not differ on gender but was greater among older adolescents (90% acceptance among 18–20 year-olds vs. 25% of 13 year-olds. The authors note that HIV testing during the three-month study period in 2009 was 446% greater than for a comparable period in 2008 (213 vs. 39 patients tested).

##### Critique

The primary achievement of this testing program was its ability to greatly increase HIV testing at this PED, as compared to prior practice. Limitations of the program include that a small fraction (11%) of eligible patients were approached for HIV testing; the responsibility of offering testing mainly fell to PED physicians, who could have forgotten or did not have time to offer testing; there no were prompts or reminders to offer testing; and written consent and counseling requirements might have impeded the number of patients who could have been tested. The analysis of this program has the common limitations of retrospective medical record reviews, including the completeness, quality and need for interpretation of the documentation available to the study.

#### Rakhmanina, et al. Journal of Adolescent Health, 2013 [17]

##### Program description

Age-eligible patients and guardians at the two PEDs in this HIV testing program were approached either during triage or in their PED room, unless the patient had a previous documented HIV test in the ED [17]. A patient was considered to opt-out if the patient and/or guardian declined screening (details of precisely how the opt-out approach was executed were not provided). The most commonly reported reasons by medical staff for not approaching an age-eligible patient included insufficient staff time, a medical decision not to address HIV screening, and the patient was already known to be HIV infected. Reasons patients cited for opting out of testing were having a recent negative HIV test, not being sexually active, and perceiving themselves of not being at risk. In a multivariable logistic regression model, greater HIV testing acceptance was associated with age ≥ 15 years-old, residing in DC, Black race, and having a parent/guardian present.

##### Critique

This program’s strengths include offering HIV testing and testing a large number of patients, utilizing rapid point-of-care testing, providing results during the patient visit, and successfully identifying and linking to care eight patients with HIV. Limitations were a lack of a clear description of the opt-out process (so that its usage can be accurately assessed) and multiple false positive tests from oral fluid sampling. Further, the opt-out approach did not prevent loss of testing opportunities (22.5% of ED visits had no testing offered) and inequalities in testing acceptance by age, race, residence, and presence of a guardian.

#### Bhatt, et al. Pediatric Quality and Safety, 2020 [18]

##### Program description

This study reported on a quality improvement initiative to overcome key barriers to increase HIV testing for patients undergoing an evaluation for STIs [18]. Improvements initiated included: (1) verbal instead of written consent for testing; (2) point-of-care HIV testing performed by ED staff instead of batched laboratory-based testing; (3) an educational campaign was implemented for PED providers to increase awareness of HIV in adolescent patients as well as the current CDC and AAP testing recommendations; (4) online HIV educational videos and informational pamphlets on HIV and STIs were available for patients to review; (5) HIV point-of-care testing order was incorporated into the STI order set (pre-selected); and (6) an automated EHR reminder posted for HIV testing when STI tests were ordered that showed the patient’s previous HIV testing and results within the last 12 months and asked the medical provider to provide a reason for not ordering testing. Documentation of HIV testing offered increased from 3.6% prior to the program initiation to 75% post-initiation and then 87% after the introduction of the automated EHR reminder.

##### Critique

This program’s strengths were its success in identifying barriers to HIV testing, implementing interventions to facilitate testing, diagnosing eight new HIV infections (over a five-year period), and its focus on sustainability. As a possible additional strength, the manuscript alludes to permitting oral fluid, fingerstick and phlebotomized blood sampling options for HIV testing, but does not specify which sampling types were actually used. Limitations include that primary outcome was offer and not performance of HIV testing, and lack of information on total eligible patients, and the number approached for, the number who accepted and the number of patients who completed testing. There were some inefficiencies noted in testing, given that there were false positive tests and testing of those previously known to be HIV infected, although linkage to care was secured for these patients.

#### Gutman, et al. Academic Emergency Medicine, 2020 [19]

##### Program description

In this pilot HIV testing program, investigators identified eligible patients through ED logs, consulted with the patient’s ED providers to confirm eligibility and assess whether the patient was already having an HIV test [19]. Testing was offered to those who were not being tested for HIV as part of their clinical care. Patients and parents or guardians were informed that the HIV test would be charged as part of their ED visit. The most commonly reported reason for declining testing (64%) was that the patient and/or guardian did not believe they were at risk for HIV. Per the results of multivariable logistic regression modeling, the presence of a parent/guardian with the patient did not affect acceptance of HIV screening (aOR 1.07, 95% CI 0.67–1.70). The study authors allude to using an opt-out approach, but do not explain how testing was offered or presented to patients. For patients who were not undergoing phlebotomy, a minimum of 400 uL of blood was collected by the study investigators via fingerstick; a blood sample was collected by the ED nurse for patients undergoing phlebotomy or who had an intravenous line in place. Only 21% of patients were notified of their test results in-person prior to discharge, and only positive or invalid results were communicated to patients after PED discharge. Negative results were only communicated in person by the screening investigator prior to PED discharge.

##### Critique

This single-site pilot HIV screening program was successful in identifying an HIV-infected younger adolescent (14 years-old) who was evaluated in the fast track area of the ED. However, this patient had a recent prior ED visit for viral syndrome during the pilot program period, but was not approached because no investigators were screening patients when the patient visited. A strength of the program was its inclusion of HIV testing costs as part of clinical care; however, there were no provisions for testing of those who were unable to pay for the costs of testing. Nearly ten percent of adolescents who opted out stated that the cost of testing was prohibitive. Limitations of this program were its short duration, selection bias in who was approached for testing (approached preferentially by chief complaint and by age, with older adolescents approached before younger patients), testing only when an investigator was present (40% of ED visits during study period, resulting in only 11% of those patients during the study period approached for testing), and that 79% of those tested did not receive their results prior to discharge. Fingerstick sample collection, although permitting more testing to occur, was also an encumbrance, given that the blood sample collection was time-consuming (reported up to 5 min) and was a large volume of blood for a fingerstick, considered to be uncomfortable by patients, and occasionally resulted in invalid results due to inadequate sample collected.

## Discussion

The eight programs in this systematic review demonstrate that HIV screening can be initiated successfully in US PEDs. As an objective marker of success, six programs identified at least one patient with an HIV infection who otherwise would have gone undiagnosed, [13–15, 17–19]. Of these, five programs found a HIV seropositivity greater than 0.1% among those tested, [13, 14, 17–19] which is the CDC recommended threshold for conducting routine screening in a given healthcare setting [4]. In further defense of HIV screening in this setting, Mollen, et al. reported that 85% of PED providers surveyed supported HIV testing in the ED and 93% approved of the program implemented in the study [14]. Two other studies apart from the eight programs reviewed in this investigation also lend support for HIV screening in EDs. Of 114 adolescents (14–21 years-old) surveyed at a Philadelphia PED, 79% agreed with the statement that “Rapid HIV testing should be included in all trips to the ED” [20]. In Mehta et al.’s survey of 191 US PEM attending physicians, 84% indicated that they believed that ED HIV screening would increase HIV testing availability to adolescents [8].

Despite these successes, the PED HIV testing programs in this systematic review highlighted challenges that limited their effectiveness at identifying HIV cases, efficiency and reach. The proportions of adolescents and young adults approached for HIV screening ranged widely, although these estimates were not reported for all programs. This variation across programs is most likely related primarily to differences in methodology across the screening programs (i.e., who, what, when, where, and how of screening) and goals of the programs (i.e., why screening). A consequent common limitation was that many patients were not approached for screening, even if intended to be “routine” or “universal”, and despite using an “opt-out” approach. Furthermore, some screening practices resulted in differences in testing based on age, race and gender.

These differences in patient selection for screening may have contributed to different HIV positivity rates across EDs. Notably, two of the sites with HIV positivity below the CDC recommended threshold for screening at 0% and 0.06%. These sites had the nontargeted approach to screening- any age-eligible patient was offered testing, as opposed to the targeted programs which selected patients deemed to have higher likelihood to have HIV given their ED presentation for possible STI. The combination of more generalized risk in the denominator of patients tested at these sites, and also low levels of testing actually offered due to low clinician compliance (older patients were more frequently offered at one site, clinicians needed to collected written consent at another site) may have contributed to the low yields. Given the low rates of offering testing at these sites, it is uncertain whether the nontargeted approach is necessarily suboptimal in the PED and merits further research.

Consent processes also were a source of variation, which in turn could have affected screening acceptance. CDC recommended removing the barrier of separate, specific written consent for HIV testing in 2006 [4]. This landmark change in recommendations for HIV testing approaches may have contributed to the increase in opt-out HIV testing seen in three of the studies this review captured after 2006. In the studies with the opt-in approach, acceptance rates were slightly lower (49.4%, 60.7%, 74.7%) compared to the opt-out approach (42.7%, 72.6%, 86.7%). The CDC consent change also may have impacted the need to obtain parental consent in certain states, though the effect of parental consent on screening acceptance is unclear. In a study conducted before the recommendation, Mehta, et al. noted that requiring parental consent resulted in some patients not being tested [13]. In contrast, Rakhmanina, et al.’s study observed that parents/guardians accompanying the patient was associated with lower odds of opting-out of HIV screening [17]. However, the investigators noted that their presence affected patient disclosure of prior HIV testing and risk-taking behavior, which might have affected HIV testing. Further, 6% of adolescents in their study who had accepted testing were not tested because parents/guardians declined it. In contrast, Gutman, et al.’s study did not find any association of HIV testing acceptance with parent/guardian presence [19].

A common source of missed testing opportunities was due to placing the onus of offering testing on PED providers, who perhaps either lacked awareness of the screening program, forgot to offer screening to eligible patients, or did not have time to offer testing. For example, Minniear, et al. reported that only 22% of PED providers surveyed prior to implementation of the screening program were aware of the revised CDC guidelines on HIV testing, and all PED providers frequently forgot to offer testing to patients, with some providers disclosing that they felt uncomfortable offering screening [15]. However, solutions to this problem are possible, as evidenced by Bhatt, et al. showing that computerized prompts in the EHR resulted in a large increase in provider ordering of HIV testing [18]. As an alternative arrangement to PED providers initiating testing, some programs reported using dedicated research assistants, [13] health educators, [14] or nurses, [15] for identifying, approaching, testing and counseling patients.

HIV testing modalities varied across the studies, partly related to the continuing evolution in tests available: phlebotomy for conventional (laboratory-based) antibody testing; [12] oral fluid sampling for conventional (laboratory-based) antibody testing; [13, 14] rapid, oral fluid, point-of-care testing; [15, 17] fingerstick sampling for rapid, point-of-care testing; [16, 18] or fingerstick or phlebotomy sampling for 4^th^ generation antigen/antibody testing [19]. The types of sampling techniques and tests employed impacted who could be tested and when tests results were received, and consequently when they could be acted upon for linkage to care. Earlier studies did not have access to the multitude of tests available currently for PED HIV testing programs, which permit not only selection based on product testing performance characteristics and price, but also according to sampling techniques, testing time, accuracy, turn-around time, and testing location (in the ED or laboratory based). Test choice in the eight studies in this review likely was decided by the funding source, and thus might not have been the optimal choice for that PED. When selection of testing was possible, there were trade-offs of ability to deploy the tests with accuracy and turn-around time.

Illustrating trade-offs in testing techniques is Gutman, et al.’s [19] program, for which blood sampling for the laboratory-based Architect HIV Ag/Ab Combo Test (Abbott Laboratories, Inc.) was either through phlebotomy or fingerstick sampling, which enabled testing for the large proportion of PED patients who did not undergo phlebotomy or have intravenous lines placed. The study authors indicated concerns about the accuracy of oral fluid sampling as a reason for using this test, as well as research suggesting that adolescents are more likely than adults to present with an acute HIV infection, which might go undetected using non-antigen tests [19]. However, a large fingerstick blood sample (400 µl) was required for this test, and sampling was performed by the study investigators, rather than ED nurses or ancillary staff. Due to the limitation of blood volume needed, 5.8% of samples had inadequate blood volumes obtained via fingerstick and could not be processed. Of the 462 patients who declined screening, 10.6% cited wanting to “avoid needles” as a reason, although the results do not indicate if this was related to phlebotomy or fingerstick sampling. Because it was a laboratory-based test with a resultant greater turnaround time than point-of-care tests, 11.7% of results were available within one hour, 63.7% within one to two hours, and 24.6% more than two hours. As a consequence, only 20.8% of patients received their results during their ED stay.

Ignoring considerations about ability to test as many patients as possible and as accurately as able, Gutman, et al.’s [19] program and others reveal how test choice affects receipt of results. Although the two early studies (Mehta, et al. [13] and Mollen, et al. [14]) used oral fluid sampling, testing was conventional or laboratory-based and not performed in rapid manner, so results were not available during the ED visit. Appointments were required to receive test results in follow-up about two weeks after the ED visit. As a consequence, Mollen, et al.’s loss to follow-up was 42% [14]. Although none of the eight studies reviewed reported being unable to secure follow-up for patients with a positive HIV test result, this problem is ubiquitous. Point-of-care rapid testing or testing with rapid results eliminate follow up appointments for receipt of test results and thus enable easier linkage to care.

The limitations of and challenges faced by these eight PED HIV screening programs can direct efforts for their implementation and for research. Feasibility no longer needs to be demonstrated. Instead, research should be directed at improving the methods of these programs, including interventions to increase their reach or coverage of patients in the PED who need to be tested for HIV, increasing testing acceptance, facilitating the performance of testing, decreasing time to test results, and ensuring patient receipt of test results. Further, optimal strategies to implement the programs in PEDs of all types commensurate with their resources and needs, as well as enabling their sustainability require attention to enable widespread and long-term success.

Potential strategies to increase PEDS implementation of HIV screening, as identified in the research covered in this review, involve leveraging provider education, the team care approach, investing in testing technology and EHR aids. Provider education should involve educational campaigns to inform providers of the prevalence of HIV in adolescents, the ability to test in the PED with linkages to care, and importantly, specific training of how to engage in conversations about sexual health. The team care approach has demonstrated success in sharing some of the time burden of additional screening and counselling conversations to members of the care team. Nurses can assist in screening questions and at certain institutions, health educators or case managers can assist in sexual health counseling and linkages to care, respectively, and lastly physician assistants and nurse practitioners can aid in follow-up calls to patients for whom positive screens result after the ED visit. As medical technology advances, the time and accessibility of point-of-care STI tests improve. Health systems' prioritization of patients’ sexual health by investing in faster rapid tests will increase the numbers of patients who can receive results during their ED visit, potentially increasing EDs willingness to incorporate HIV screening programs. Finally, the EHR can facilitate HIV screens by choice architecture of how the clinician interacts with HER by incorporating HIV screening in STI order sets as a pre-selected choice, reminding providers who do not order HIV that it can be order. Together these strategies to improve HIV screening merit further implementation science-informed research as to their effectiveness.

### Limitations

As true for this type of investigation, this systematic review is limited by what has been published on this topic. Most of the studies were demonstration or pilot programs of a short duration. This limitation therefore precluded any long-term analysis on their effectiveness and sustainability. Furthermore, most programs were single-center studies that were highly heterogeneous in design and methodology. As a result, only a qualitative review of the studies could be conducted. All articles were analyzed from the lens of current-day knowledge of HIV testing and practices, and are therefore reflect the biases, knowledge and perspectives of the reviewers. In addition, all studies were focused in the United States, and findings may not apply to testing being performed in other countries. Although we performed manual searches of journal websites in addition to our database searches, there likely were journals we either did not search or failed to record that we had searched them. The former concern might have resulted in missed articles, although the likelihood of this occurrence is small given the database search. This potential effect on the analysis cannot be known.

## Conclusion

In this systematic review, we report on US PED HIV screening programs which demonstrate their feasibility to be initiated, and ability to identify HIV-infected adolescents and link them to care. The studies illustrate opportunities to further optimize the integration of HIV screening programs within PEDs and reduce barriers to testing, improve efficiency and increase effectiveness. Future research should focus on advancing the methodology of screening programs as well as their implementation to other settings and their longer-term sustainability.

## Supplementary Information

**Additional file 1:Supplement**. Listing of journals manually searched for relevant articles. The supplement provides a list of emergency medicine, pediatric and adolescent health, and HIV journals whose websites were searched for additional relevant articles for the systematic review.

## Data Availability

All data generated or analyzed during this study are included in this published article.

## Abbreviations

CDC: Centers for Disease Control and Prevention
ED: Emergency department
EHR: Electronic health record
HIV: Human immunodeficiency virus
PED: Pediatric emergency department
PEM: Pediatric emergency medicine
PRISMA: Preferred Reporting Items for Systematic Reviews and Meta-Analyses
STI: Sexually transmitted infection/disease
US: United States
USPSTF: United States Preventive Services Task Force
