# Supplementary material for: A systematic review of HIV screening programs conducted in pediatric emergency departments in the United States
Source: BMC Emerg Med. 2022 May 6;22:75. doi: 10.1186/s12873-022-00633-5 (PMC9074268; doi:10.1186/s12873-022-00633-5)
Supplement: Supplementary file 1 — Additional file 1:Supplement. Listing of journals manually searched for relevant articles. The supplement provides a list of emergency medicine, pediatric and adolescent health, and HIV journals whose websites were searched for additional relevant articles for the systematic review. [file 12873_2022_633_MOESM1_ESM.docx]

| **Supplement: listing of journals manually searched for relevant articles**  **Emergency medicine journals** |
| --- |
| Academic Emergency Medicine  American Journal of Emergency Medicine  Annals of Emergency Medicine  Australasian Emergency Care  BMC Emergency Medicine  California Journal of Emergency Medicine  Canadian Journal of Emergency Medicine  Critical Care Research and Practice  Emergency Medicine Journal  Journal of the American College of Emergency Physicians Open  Pediatric Emergency Care  Western Journal of Emergency Medicine |

| **Pediatric and adolescent health journals**  Academic Pediatrics  Advances in Pediatrics  BMC Pediatrics  Clinical Pediatrics  [Comprehensive Child and Adolescent Nursing](https://www.ncbi.nlm.nih.gov/nlmcatalog/101682864)  [Current Pediatric Reviews](https://www.ncbi.nlm.nih.gov/nlmcatalog/101240290)  [Current Problems in Pediatric and Adolescent Health Care](https://www.ncbi.nlm.nih.gov/nlmcatalog/101134613)  European Journal of Pediatrics  [Hospital Pediatrics](https://www.ncbi.nlm.nih.gov/nlmcatalog/101585349)  International Journal of Adolescent Medicine and Health  I[talian Journal of Pediatrics](https://www.ncbi.nlm.nih.gov/nlmcatalog/101510759)  Journal of Adolescent  The Journal of Adolescent Health  [JAMA Pediatrics](https://www.ncbi.nlm.nih.gov/nlmcatalog/101589544)  Journal of Pediatric Health Care  Journal of the Pediatric Infectious Diseases Society  Journal of Research on Adolescence  Journal of Youth and Adolescence  The Lancet, Child and Adolescent Health  Pediatric Annals  Paediatrics and International Child Health  The Pediatric Infectious Disease Journal  Pediatric Research  Pediatrics in Review |  |
| --- | --- |
| **HIV journals**  AIDS Patient Care and STDs  Current HIV/AIDS Reports  Current HIV Research  Current Opinion in HIV and AIDS  HIV Medicine  HIV Research and Clinical Practice  [HIV research & clinical practice](https://www.ncbi.nlm.nih.gov/nlmcatalog/101738312)  Journal of the International AIDS Society  The Lancet HIV  Sexual Health | |
